# A prospective study of midfoot osteotomy combined with adjacent joint sparing internal fixation in treatment of rigid pes cavus deformity

**DOI:** 10.1186/1749-799X-9-44

**Published:** 2014-06-05

**Authors:** You Zhou, Binghua Zhou, Junpeng Liu, Xiaokang Tan, Xu Tao, Wan Chen, Kanglai Tang

**Affiliations:** 1 Department of Orthopaedics, Southwest Hospital, Third Military Medical University, No.30 Gaotanyan Road, Chongqing 400038, People's Republic of China

**Keywords:** Pes cavus, Midfoot osteotomy, Treatment, Adjacent joint sparing internal fixation

## Abstract

**Background:**

Midfoot osteotomy has been previously confirmed to be a good method to correct pes cavus. How to fix the osteotomy and which point to choose for the procedure has been a focus for most surgeons. The aim of this study was to analyse the outcomes of a series of patients who had been treated for pes cavus deformity using midfoot osteotomy combined with adjacent joint sparing internal fixation.

**Materials and methods:**

Between 2008 and 2012, 17 patients with a mean age of 16.8 years (12–36 years) were tracked after treatment by midfoot osteotomy combined with adjacent joint sparing internal fixation with three cannulated screws between the Lisfranc line and Cyma line. Clinical outcomes were assessed by measuring improvements of appearance and function, American Orthopedic Foot and Ankle Society (AOFAS) scores, and radiographic changes.

**Results:**

The mean follow-up time was 25.3 months (range, 10–50). The mean healing time from the osteotomy was 7.8 weeks (range, 6–12). The appearance and weight-bearing function were significantly improved in all patients. At a final follow-up, the mean AOFAS score was 75.8/100 points (range, 63–90). The mean Meary's angle, calcaneal pitch angle, tibiotalar angle, and Hibb's angle values improved from 26.3 to 5.5, 44.5 to 28.3, 133.1 to 100.8 and 66.9 to 41.1, respectively. Adjacent joints presented no obviously arthritic degeneration at the follow-up. Subjectively, 94.1% of patients were very satisfied or satisfied with minor reservations. Objective outcomes were excellent or good in 88.2% of feet.

**Conclusion:**

For the treatment of rigid pes cavus deformity, extra-articular midfoot osteotomy combined with adjacent joint sparing internal fixation is effective and safe. This surgical technique is especially effective with low rates of arthritic degeneration and joint stiffness in the adjacent joints and little reduction of ankle and foot flexibility.

## Background

Pes cavus is a common foot deformity characterised by an abnormally high arch medially in the sagittal plane of the foot. It may be accompanied by additional deformities of the forefoot or hindfoot. Pes cavus occurs mostly secondary to neuromuscular diseases, but it can result from trauma or even from idiopathic or congenital abnormalities [1,2]. Pes cavus can change the shape of the foot, the patient's gait, and the stability of the ankle, which seriously affects the patient's weight-bearing and walking functions [3,4]. Conservative treatments are often used to treat mild flexible cavus. For rigid cavus, surgical interventions are needed.

Midfoot osteotomy, first described by Cole [5], has been shown to have a good success rate for correcting pes cavus in recent years. However, most surgeons have focused solely on how to fix the osteotomy and at which point the osteotomy should be performed. To achieve rigid immobilisation, the adjacent joints are fixed in most osteotomy procedures, by either internal or assisted external fixation. Furthermore, the apex of the deformity is often located where surgeons choose to perform the osteotomy, which passes through the midfoot joints and destroys the articular surface. As a result, some complications cannot be avoided, including the degeneration of adjacent joints, joint stiffness and reduction of ankle and foot flexibility [5,6].

To reduce complications, we proposed performing midfoot osteotomy extra-articularly and using an adjacent joint sparing internal fixation technique to treat pes cavus. The aim of this study was to analyse the outcomes of a series of patients who had been treated for pes cavus deformity using midfoot extra-articular osteotomy combined with adjacent joint sparing internal fixation.

### Patients and methods

This was a prospective study of all available patients. From 2008 to 2012, a series of patients with rigid pes cavus were treated surgically at our department using midfoot osteotomy combined with adjacent joint sparing internal fixation. The patients underwent surgery for deformity, pain or to correct gait abnormalities and reconstruct the stability of the foot and ankle. All surgeries were performed by two experienced orthopaedic surgeons at a single institution. Exclusion criteria included patients with flexible pes cavus or with severe deformities or serious osteoarthritis complications.

We initially enrolled 20 patients, but two were excluded because they needed additional surgery, one for serious osteoarthritis of the subtalar joint, the other for serious hindfoot varus; another patient was excluded because he was lost to follow-up. Therefore, the final study included 17 patients after obtaining informed consent from all of them and the approval of the Hospital Ethics Committee (Southwest Hospital affiliated to Third Military Medical University in China). Our research on humans was in compliance with the Helsinki Declaration.

The mean age at surgery was 16.8 years (12–36 years); ten patients were male, and seven were female. Eight patients had surgery on their left foot, nine on the right foot. The aetiology was poliomyelitis in ten cases, spinal cord tumour in one case, and sequela of trauma in two cases. Four patients presented with idiopathic pes cavus.

**Figure 1:**
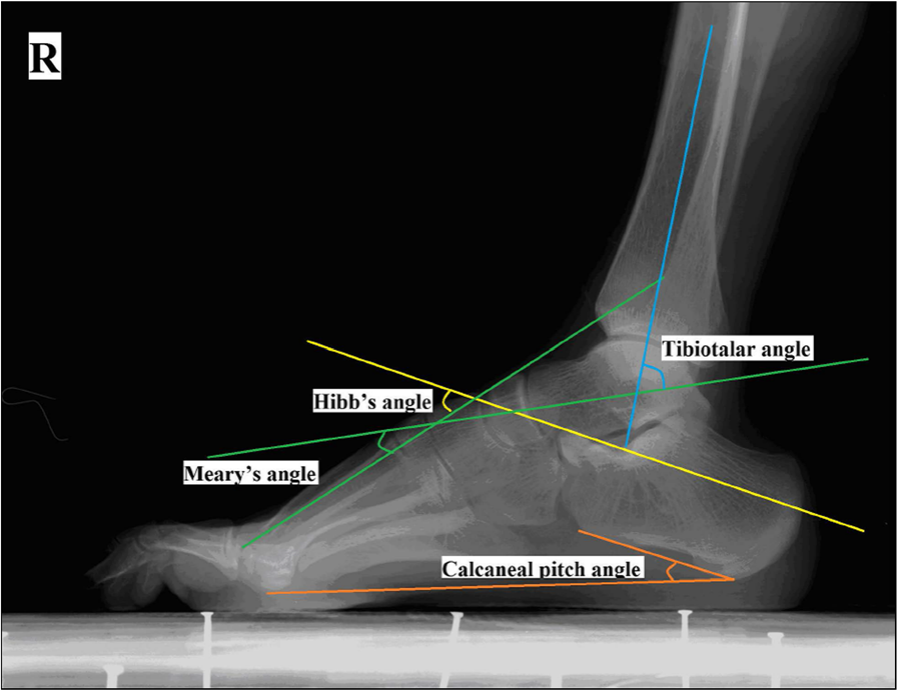
**Preoperative plan.** Measurement of Meary's angle, calcaneal pitch angle, tibiotalar angle and Hibb's angle.

The clinical evaluation consisted of two parts: subjective and objective assessments. The subjective assessment evaluated a patients' pain, foot appearance, shoe fitting and weight-bearing. The objective assessment measured American Orthopedic Foot and Ankle Society (AOFAS) foot scores, Japas' criteria [7] and radiographic results (Meary's angle, calcaneal pitch angle, tibio-tarsal angle and Hibb's angle) (Figure 1). The osteoarthritis of adjacent joints was assigned, according to Morrey-Weidman's standard [8], as stage 0 (normal joint), stage I (moderate joint space narrowing and osteophytosis), stage II (distinct joint space narrowing, subchondral condensation and edge sclerosis) or stage III (severe arthritis).

Statistical analysis was performed with SPSS 13.0 software (version 13.0; SPSS Inc., Chicago, IL, USA), using Student's paired *t* test to compare the means. The level of statistical significance was considered at 5% (*P* < 0.05).

### Surgical technique

**Figure 2:**
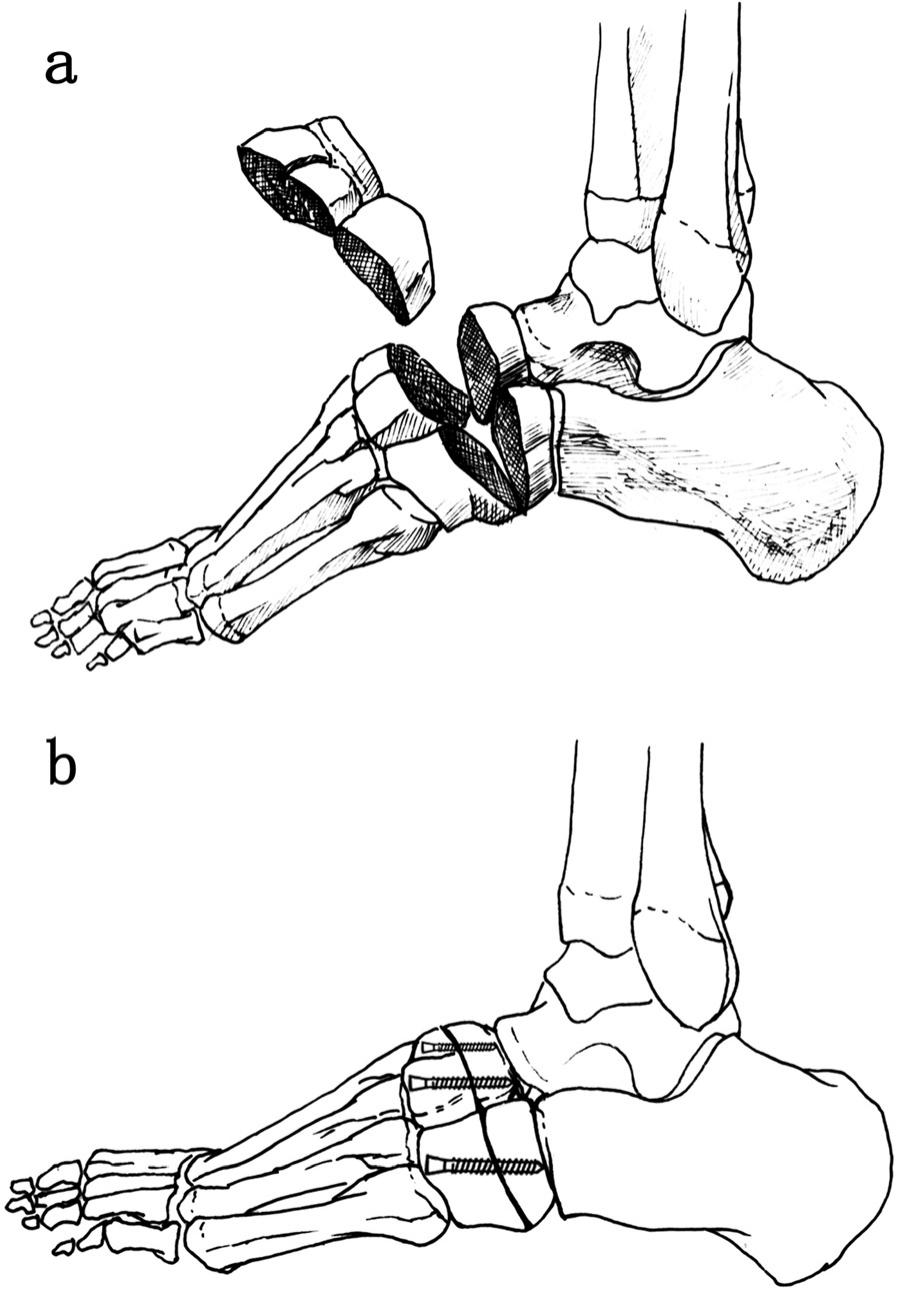
**Surgical technique. (a)** A wedge-shaped osteotomy was performed, including part of the cuneiform, cuboid and navicular, just proximal to the first and fifth tarso-metatarsal joints. **(b)** Three cannulated screws along the K-wires were applied to fix the osteotomy.

**Figure 3:**
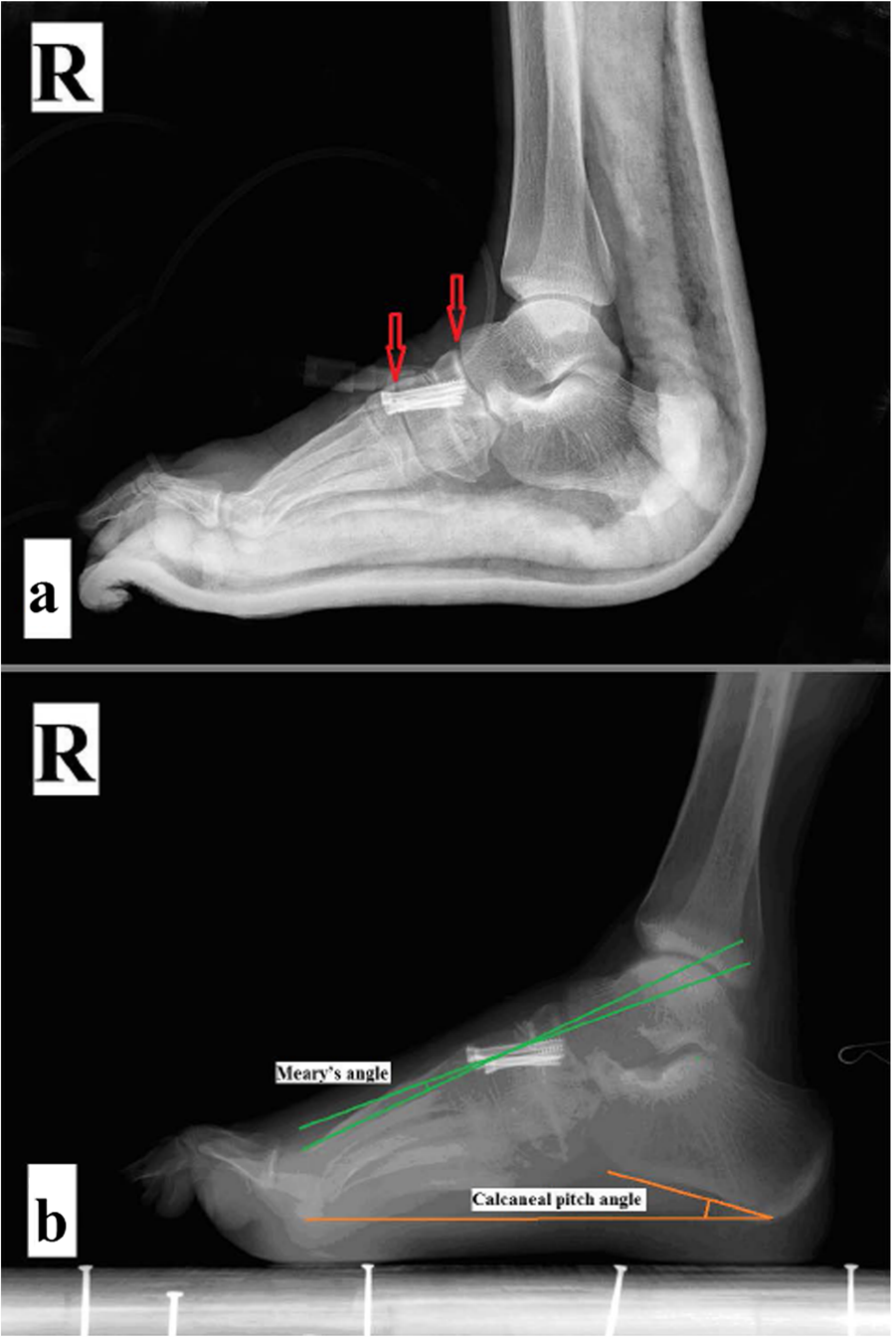
**Postoperative X-ray. (a)** The adjacent joint sparing internal fixation between the Lisfranc line and the Cyma line (arrow) is shown. **(b)** Bony union of the midfoot osteotomy and no obvious degeneration of adjacent joints 6 months postoperatively is shown.

Surgery was performed under epidural anaesthesia. The patient was placed in a supine position, with a pad under the ipsilateral buttock and a tourniquet placed around the proximal thigh. First, a percutaneous release of the plantar fascia was performed through a medial short incision. Second, a 6-cm longitudinal incision was made in the middle of the dorsum of the foot. The cuneiforms, cuboid and navicular were exposed by sharply separating the gap between the second and third digital extensor tendons. A wedge-shaped osteotomy was performed, including part of the cuneiform, cuboid, and navicular, just proximal to the first and fifth tarso-metatarsal joints (Figure 2a). The distal part of the foot was then pulled distally and compressed at the osteotomy site. Three K-wires were passed distal to proximal to fix the osteotomy temporarily. Then, three cannulated screws (from Newdeal, New York, NY, USA; diameter: 4.3 mm) were applied along the K-wires to permanently fix the osteotomy (Figures 2b, 3a) strictly limited between the Lisfranc line and Cyma line, which was confirmed by intraoperative fluoroscopy.

### Additional operations

The posterior tibial tendon was transferred selectively in some patients through the interosseous membrane to the dorsum of the foot using a suture anchor. In those cases where Achilles tendons were contractural, Z-plasty of the Achilles tendon was performed percutaneously. When necessary, the claw toes were immobilised in extension with K-wires.

### Postsurgery managements and evaluation

A below-knee cast was applied for 4 weeks. Then, the patients were instructed on active mobilisation exercises of the foot and ankle and partial weight-bearing with the protection of cast. K-wires used for immobilising the claw toes were removed 6 weeks postoperatively, and the cannulated screws were removed 3 to 6 months postoperatively when the osteotomy was confirmed by radiograph to have reached bony union.

### Follow-up

All patients were given outpatient reviews at 4 weeks, 6 weeks, 12 weeks, 6 months and 1 year postoperatively. After bony union of the osteotomy, the follow-up interval was 1 year.

## Results

The mean follow-up time was 25.3 months (range, 12–48). There were no major complications, such as infection, skin necrosis, vascular or nerve injuries or failure of internal fixation. Midfoot osteotomies reached bony union in all patients, and the mean healing time was 7.8 weeks (range, 6–12) (Figure 3b). Two patients had foot pain located in the head of the metatarsal and the midfoot. Improvement of foot appearance and gait was found in all patients, and 94.1% (16/17) of patients were very satisfied or satisfied with minor reservations. Three reported using loose shoes because of pain or worries about the recurrence of the deformity.

**Figure 4:**
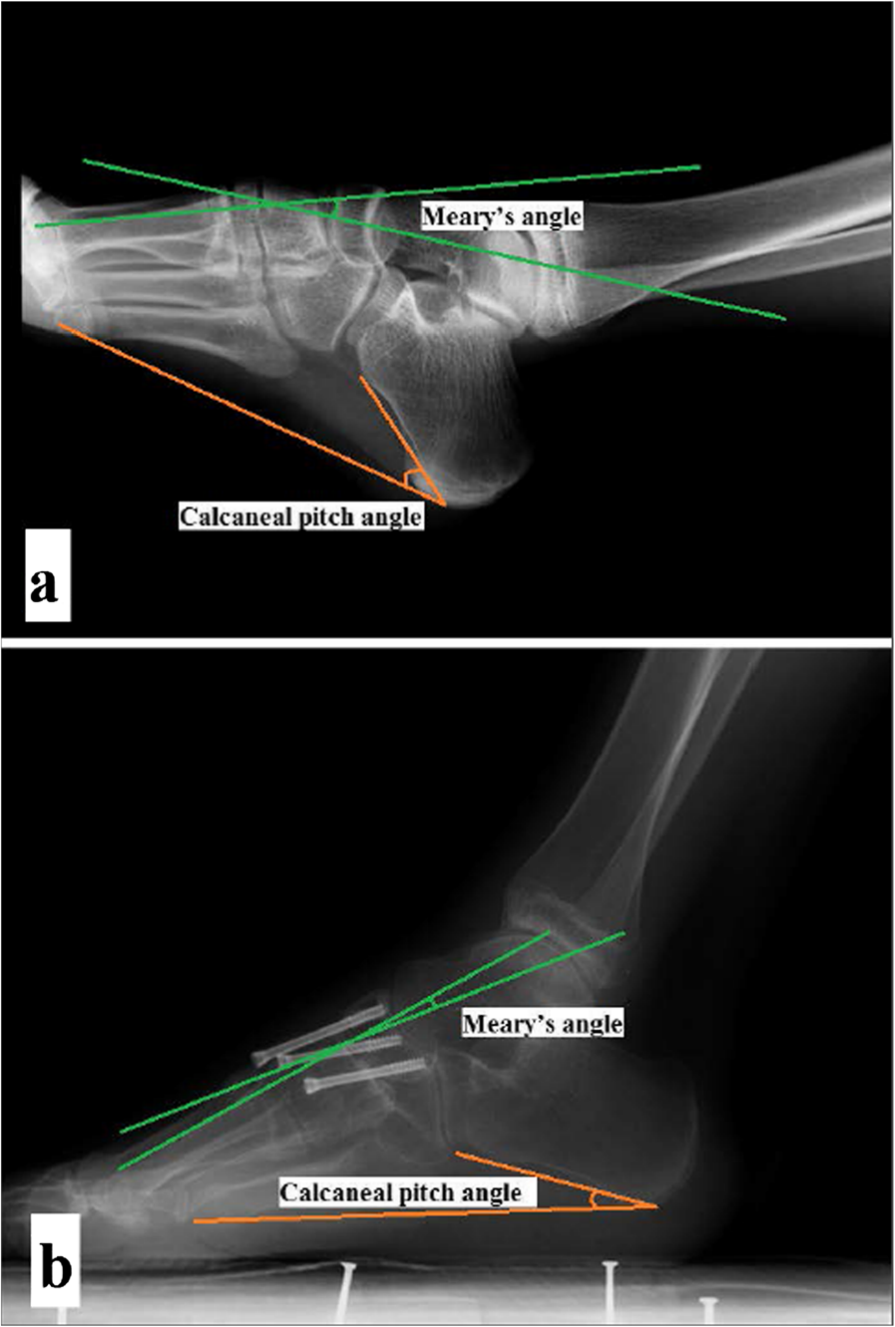
**A severe deformity case achieved good correction.** A lateral X-ray of a foot **(a)** shows the preoperative deformity, and **(b)** a postoperative X-ray of the same patient after midfoot osteotomy and adjacent joint sparing internal fixation is shown.

At the final follow-up, the mean AOFAS score was 75.8/100 point (range, 63–90), which was significantly higher than the 34.7/100 point score measured preoperation (Table 1). Meary's angle, the calcaneal pitch angle, the tibiotalar angle and the Hibb's angle improved significantly, from 26.3 (5.7) to 5.5 (2.8), 44.5 (5.7) to 28.3 (3.3), 133.1 (6.9) to 100.8 (5.6), and 66.9 (8.3) to 41.1 (4.3), respectively (Table 2). According to Japas' criteria (Table 3), we had very good results in 11 cases (64.7%) and good results in 4 cases (23.5%) (Figure 4). Osteoarthritis (stage I–II) of the subtalar joint existed in four preoperative cases; no deterioration was found postoperatively. Others found no osteoarthritis in adjacent joints.

**Table 1:** AOFAS scores: preoperative versus postoperative

	**Preoperative**	**Final follow-up**	***P *** **value**
	**Mean (SD)**	**Mean (SD)**	
AOFAS scores	34.7(6.2)	75.8(8.3)	0.000*

*Statistically significant results, *p* < 0.05.

**Table 2:** Radiographic result: preoperative versus postoperative

	**Preoperative**	**Final follow-up**	***P *** **value**
	**Mean (SD)**	**Mean (SD)**	
Meary's angle (°)	26.3(5.7)	5.5(2.8)	0.000*
Calcaneal pitch angle (°)	44.5(5.7)	28.3(3.3)	0.000*
Tibiotalar angle (°)	133.1(6.9)	100.8(5.6)	0.000*
Hibb's angle (°)	66.9(8.3)	41.1(4.3)	0.000*

*Statistically significant results, *p* < 0.05.

**Table 3:** **Japas' criteria**[7]

**Japas' criteria**	
Very good	Complete correction of the deformity; painless gait and full movement at the subtalar and midtarsal joints
Good	Incomplete or partial correction of deformity and some pain at the metatarsal heads during walking

## Discussion

Pes cavus is characterised by a high arch in the sagittal plane of the foot [9-11]. Its clinical manifestations include steppage gait, midfoot pain, pain and ulceration in the head of metatarsal, joint stiffness, instability of the lateral ankle, and other symptoms [12-14]. The surgical correction of pes cavus is intended to eliminate pain, restore plantigrade foot confirmation and recover the balance of bone alignment, muscle forces and joint mobility [2,15,16]. Previous surgical treatments include soft tissue releases, tendon transfers, various osteotomies, arthrodeses and so on [17-19]. The surgical management should be chosen according to the type and severity of deformity.

Midfoot osteotomy was first proposed by Cole in 1940 [5] and has achieved considerable curative results in the past decades. Tullis et al. [20] reported a 100% fusion rate in eight patients (11 feet) who underwent the Cole osteotomy. Levitt et al. [21] reported a 30% pseudarthrosis rate with use of the Cole osteotomy. Naudi et al. [22] reviewed 33 patients (39 feet) over a long-term follow-up period. They found that 70% were satisfied with their treatment results. They also found that 74.2% had arthritic changes in the subtalar joint (18 feet) and tarsometatarsal joints (17 feet), and they believed that the correction capacity of anterior tarsectomy was limited. For severe deformities, bone resection treatment would be either insufficient or excessive and could lead to mediotarsal joint space arthritis, so severe pes cavus cases were excluded from the present study. Although midfoot osteotomy has been confirmed to be effective for the treatment of pes cavus, there are still considerable complications, especially degeneration of the adjacent joints and pain and dysfunction of the ankle and foot [22,23]. In our results, no postoperative deterioration was found in the osteoarthritis of the adjacent joints; in all cases, no obvious decrease of the foot and ankle mobility was found.

The usual methods of internal fixation include K-wire and stapling, both of which are not reliable enough to permit early functional exercises [24,25]. A different internal fixation tool, the cannulated screw, is believed to be a good choice to compress the osteotomy site. However, to acquire a rigid fixation, the screw must cross the adjacent joints, such as the talonavicular joint, the calcaneocuboid joint, the tarsometatarsal joint and the subtalar joint, which are all amphiarthrodial joints. Therefore, early exercise is likely to result in pain, injury to the articular cartilage and internal fixation rupture. To avoid the above complications, we applied three cannulated screws to fix the osteotomy between the Lisfranc line and the Cyma line. Our method permitted early exercise, which was beneficial to the recovery of joint function. Follow-up results of our 17 study cases demonstrated that midfoot osteotomies formed bony unions in all patients, and the improvements in AOFAS scores and radiographic results were significant. Complications such as degeneration of the adjacent joints, internal fixation rupture and dysfunction of the ankle and foot were successfully avoided.

Using adjacent joint sparing fixation in fixing the midfoot osteotomy is more challenging than using previous fixation techniques. If the screws are too short, the fixation will be not reliable; if the screws are too long and exceed the joint line, it will defeat the purpose of adjacent joint sparing fixation. Therefore, this operation should be carried out under fluoroscopic guidance, and the surgeon should strictly control the entry point, direction and length of the screw.

The present study had a few limitations. First, the mean duration of follow-up was limited to 25.3 months. Therefore, to evaluate the long-term outcomes of our technique for the treatment of pes cavus deformity, this cohort will need to be reassessed after a longer follow-up period. Second, not all patients were managed uniformly. Some underwent posterior tibial tendon transposition and/or Z-plasty of the Achilles tendon. While these additional operations have had little influence on the outcome parameters of the present study, it is not possible to distinguish the independent benefits.

## Conclusion

We conclude that extra-articular midfoot osteotomy, combined with adjacent joint sparing internal fixation, is effective and safe for the treatment of rigid pes cavus deformity. This surgical technique is especially effective with low rates of arthritic degeneration and joint stiffness in the adjacent joints and little reduction of ankle and foot flexibility, providing patients with good clinical outcomes.

## Competing interests

The authors declare that they have no competing interests.

## Authors’ contributions

YZ participated in collecting data and drafting the manuscript. BZ participated in collecting data. JL participated in statistics and in collecting data. XT (Tan) participated in surgery and evaluation. XT (Tao) participated in the study design. WC participated in evaluation. KT participated in the study design and surgery and final approval. All authors read and approved the final manuscript.
